# Frequency difference beyond behavioral limen reflected by frequency following response of human auditory Brainstem

**DOI:** 10.1186/1475-925X-13-114

**Published:** 2014-08-09

**Authors:** Qin Xu, Qin Gong

**Affiliations:** 1 Postal address: Department of Biomedical Engineering, Medical School, Tsinghua University, Beijing 100084, China

**Keywords:** Frequency following response (FFR), Frequency difference limen (FDL), Frequency discrimination

## Abstract

**Background:**

The present study investigated whether the frequency-following response (FFR) of the auditory brainstem can represent individual frequency-discrimination ability.

**Method:**

We measured behavioral frequency-difference limens (FDLs) in normal hearing young adults. Then FFRs were evoked by two pure tones, whose frequency difference was no larger than behavioral FDL. Discrimination of FFRs to individual frequencies was conducted as the neural representation of stimulus frequency difference. Participants were 15 Chinese college students (ages 19–25; 3 males, 12 females) with normal hearing characteristics.

**Results:**

According to discriminative neural representations of individual frequencies, FFRs accurately reflected individual FDLs and detected stimulus-frequency differences smaller than behavioral threshold (e.g., 75% of FDL).

**Conclusions:**

These results suggest that when a frequency difference cannot be behaviorally distinguished, there is still a possibility of it being detected physiologically.

## Background

Pitch plays an important role in the perception of speech, language and music. In general, sounds may be ordered on a scale extending from low to high pitch. In speech, pitch carries information about talker identification and emotions. In music, changes in pitch convey melody. For pure tones, the physical correlate of pitch is frequency [1]. By convention, the pitches of pure tones are used as standards to judge the pitches of other sounds [2].

The electrical frequency-following response (FFR) recordable from the scalp reflects the sum of sustained phase-locked activity evoked by periodic sounds in brainstem neurons [3,4]. Stimulus periodicity (and hence frequency) is reflected in the FFR, which includes pitch-relevant information [4,5] allowing, for instance, to track the pitch contours of the four lexical tones of Mandarin Chinese [5,6]. Thus, FFR is related to behavioral pitch perception. Deficient phase-locking of FFR is reported for elder people [7,8] and for children with autism spectrum disorders [9]. Tone-language speakers [6,10-12] and musicians [13-15] produce stronger FFRs than English speakers or non-musicians. Appropriately, higher FFR pitch-tracking accuracy accompanies improved behavioral performance following training [16,17]. Specifically, correlation between behavioral pitch discrimination and FFR has been investigated in previous studies. FFR strength covaries with fundamental-frequency difference limens (F0DLs) for iterated rippled noise with increasing temporal regularity [18,19]. For the detection of unresolved harmonic complex tones in noise, increased F0DLs and decreased FFR strength correlate with increases in noise level [20]. In the case of musical pitch, a significant association between FFR representation of F0 and behavioral F0DL is found for musicians (but not for non-musicians or tone-language speakers [15]). Short-term training improves both behavioral measures of pitch discrimination and FFR strength for complex tones with rising or static pitch contours (but not for falling pitch contours) [17]. A significant correlation is found between FFR strength and behavioral frequency difference limen (FDL) for pure tone in one study [21] but not in another [8].

The aforementioned studies focused on the relationship between FFR phase locking to a single frequency and behavioral pitch discrimination. The present study explores the FFR representations of frequency discrimination more directly. Specifically, FFRs evoked by two tones with different frequencies were discriminated so as to represent the frequency difference. We ask how well FFRs evoked by two frequencies can detect their frequency difference. Frequency discrimination abilities of normal hearing subjects were assessed. In each subject, the smallest detectable frequency difference between two pure tones, the frequency-difference limen (FDL), was measured psychoacoustically. Then we investigated whether FFR can detect frequency differences equal to, or even smaller than, the behavioral FDLs.

## Methods

### Experiments arrangement

Behavioral FDLs were obtained first for all subjects. Then, FFRs for two-tone stimuli were recorded for tones with frequency difference equal to: 1) FDL (100% FDL condition); 2) 75% of FDL (75% FDL condition); and 3) 50% of FDL (50% FDL condition). Some subjects with relatively large FDLs (with 50% FDL similar to the 75% FDL of other subjects) were selected to participate in the 50% FDL condition. The 50% FDLs of these subjects were paired with the 75% FDLs of others. Control FFRs were recorded with the earphone blocked and the subject’s ear occluded. The order of the FFR recordings was randomized. Each test or condition lasted around half an hour. For each subject, the tests were taken separately on different days in two weeks.

### Subjects

FDLs were obtained from fifteen college students (3 males, 12 females; ages 19–25). Their FFRs were then recorded in the 100% and 75% FDL conditions. Six of the subjects were additionally tested in the 50% FDL condition. All subjects were native speakers of Mandarin Chinese and had normal hearing sensitivity (better than 15 dB HL for octave frequencies from 250 to 8000 Hz). Participants reported no history of neurological or psychiatric illnesses, and no music instruction. All subjects were paid for their time and gave informed consent in compliance with a protocol approved by the institutional review board at Tsinghua University.

### Psychoacoustic experiment

#### *Stimuli*

Stimuli were tone pairs. One tone was always set at 140 Hz (the reference frequency or F_ref) and the other (the comparison frequency or F_comp) at a higher frequency. The duration of both tones was 250 ms, including 10-ms rise/fall times shaped with a Blackman window. An insert earphone delivered the stimuli to the right ear. The overall level of each tone including onset and offset ramps was 83 dB SPL. Calibration was performed with a Brüel & Kjær type 3160-A-042 sound analyzer and a type 4157 2-cc coupler.

#### *Behavioral procedure*

Frequency discrimination was tested using an adaptive three-interval forced choice procedure [two-down, one-up rule [22], programmed using MATLAB]. For each test trial, subjects heard three sequential intervals, two of them identical, containing the reference frequency F_ref, and the other one containing the comparison frequency F_comp. F_comp was always higher than 140 Hz and its initial value was 170 Hz. The three tones for each trial were assigned randomly. Subjects were instructed to identify the interval perceived as having a higher pitch by mouse clicking on the corresponding button on a computer monitor. The inter-sound interval was 0.8 s and there was a pause with 3-s duration between the subject selecting an answer and the beginning of the next trial. After two consecutive correct responses, F_comp was decreased by one step for the subsequent trial; conversely, F_comp was increased by one step following a single incorrect response. The step size was changed adaptively: its initial value, 6 Hz, was decreased to $1/\sqrt{2}$ of its previous value after each reversal. When the step size reached a pre-determined small value (0.1 Hz), it remained fixed. Each trial included 14 reversals, and the geometric mean of the frequency differences (F_comp-F_ref) across the last 8 reversals was taken as the FDL (in Hz). Every subject took at least one practice test and two formal tests. The mean of the formal tests was taken as the individual’s behavioral FDL. All tests were performed in an acoustically- and electrically-shielded booth.

### FFR experiment

#### *Stimuli*

Two tones with frequencies F_ref and F_comp were presented alternatively. The duration of each tone was 144 ms, including 7-ms rise/fall times shaped with a Blackman window and 0° initial phase. This duration, shorter than that used in the psychoacoustic experiment, was of little consequence, since frequency discrimination improves minimally with increasing duration beyond 100 ms [23,24]. Stimuli were delivered to the right ear at 83 dB SPL through an insert earphone (Etymotic, ER-2) at a rate of 2.4 per second. The earphone was shielded in a Faraday cage, with the transducers and electric wires wrapped by aluminum foil linked to common ground [25,26].

F_ref was always 140 Hz but F_comp varied across the three FFR conditions for each subject. 1) In the 100% FDL condition, F_comp equaled 140 Hz plus FDL. 2) In the 75% FDL condition, F_comp equaled 140 Hz plus 75% of FDL. 3) In the 50% FDL condition, F_comp equaled 140 Hz plus 50% of FDL.

#### *FFR recording*

**Figure 1:**
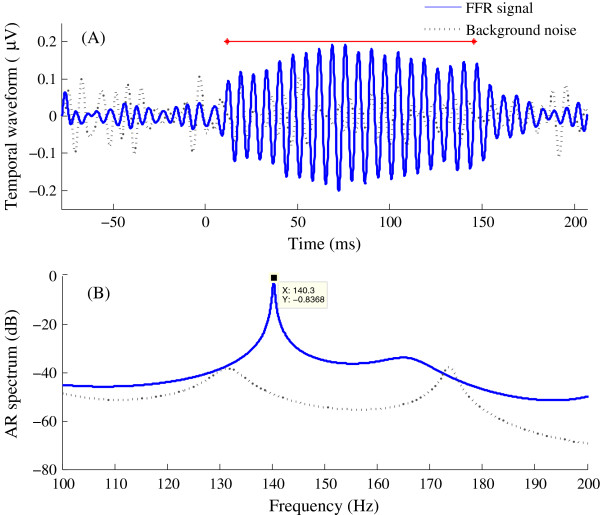
**FFR temporal waveform and AR spectrum.** Data are for a subject with FDL of 5.63 Hz. The solid lines show the time-domain waveform **(A)** and the AR spectrum of the FFR evoked by F_ref (140 Hz) in the 100% FDL condition **(B)**. The interval 12 ~ 145.6 ms subjected to spectrum analysis is indicated by the red solid line in panel A. Noise floors are indicated by dotted lines. The background noise was recorded and analyzed in the same way as the FFR, except that the earphone was blocked to prevent the stimulus from reaching the eardrum.

Subjects were seated comfortably in an acoustically- and electrically-shielded booth. They were instructed to relax and to refrain from moving during data recording to minimize myogenic artifacts. FFRs were recorded with a Grass LP511 AC amplifier and digitized by a National Instruments data acquisition card, as described in our previous work [27]. A vertical electrode montage [3,4], with the non-inverting electrode placed on the midline of the forehead at the hairline (+, F_z_), a reference electrode placed on the ipsilateral mastoid (-, M2) and the common ground electrode placed on mid-forehead (F_pz_). FFRs were recorded differentially from F_z_-to-ipsilateral mastoid, amplified by a factor of 50,000 and band-pass filtered (1–3000 Hz) online. FFR recording was started 77.7 ms before the onset of stimuli and was ended at 61.6 ms post-stimuli offset. Two thousand sweeps were recorded for each tone with a sampling rate of 10,000 Hz. The ear probe included an ER-2 earphone and a microphone (ER-10B+, Etymotic research), through which the ear-canal sound pressure was recorded concurrently with FFR recordings. The sound pressure signal was used to ascertain appropriate placement of the earphone and to measure the system delay, including the travel time in the 30-cm rubber tubes. In addition, the cross-correlation between the sound pressure signal and FFR recording was performed and the time lag corresponding to the highest peak of the cross-correlation function was taken as the latency of FFR. All the latencies of our FFR recordings were in the range of 5 ~ 10 ms, much larger than the latency of cochlear microphonic occurring within 1 ms after the stimulation onset [28]. Background EEG noise was also recorded when stimuli were rendered inaudible by blocking the earphone. As shown by the dotted line in Figure 1, there were no periodic components in the background noise. Thus, our FFR recordings reflected neural activity rather than CM or stimulus artifact [27].

### FFR data processing and analysis

FFR processing and analysis were done offline, using software programmed in MATLAB, after isolating the 2000-sweep raw data for each of the two tones. Pre-processing was done first and then data were selected according to the calculated SNR. FFRs were analyzed both in the frequency and time domains (using spectra and autocorrelations, respectively) to extract the frequency encoded in the FFR. To represent the stimulus frequency difference, FFRs to paired tones was discriminated by comparing their spectra and autocorrelations.

#### *Pre-processing*

Details about pre-processing were described in our previous work [27]. Monitoring of sound in the ear canal permitted the identification and exclusion of trials which were contaminated by subject motion or slippage of the earphone. Trials in which the FFR signal excursions exceeded 95% of the measuring range of the recording equipment were also excluded from further analysis. The remaining trials were averaged together. Then, a posterior Wiener filtering and band-pass (70–210 Hz) filtering were used to reduce noise. The analysis time window was 12 ~ 145.6 ms, corresponding to the steady-state portion of the stimulus tones.

#### *Signal-to-noise ratio (SNR)*

After pre-processing, SNRs were calculated taking the intervals 12 ~ 145.6 ms and -77.7 ~ 0 ms, respectively, as the signal and the noise. The SNR is the ratio of the root mean square (RMS) amplitude of the FFR signal relative to the RMS amplitude of the noise, expressed in decibels. In cases where the FFR was smaller than the noise (i.e., SNR < 0 dB), the subject’s FFR data were excluded from further statistical analysis. This happened for one subject in the 100% FDL condition and another in the 75% FDL condition. After these exclusions, the SNR values were 8.66 ± 4.1 dB (mean ± standard deviation) for the 100% FDL condition and 8.31 ± 2.78 Hz for the 75% FDL condition.

#### *Spectrum analysis*

**Figure 2:**
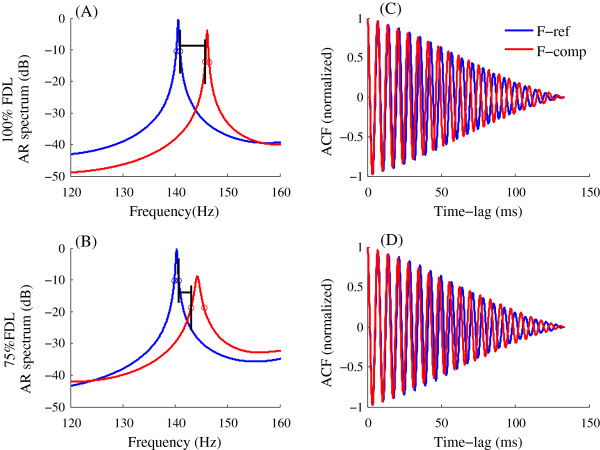
**Spectra and ACFs of FFRs evoked by two frequencies.** Data are from a subject with FDL of 5.63 Hz. Panels in the left **(A, B)** show the AR spectra and panels in the right **(C, D)** show the corresponding ACFs. Top and bottom panels illustrate results for the 100% and 75% FDL conditions, respectively. Blue and red lines indicate FFRs evoked by F-ref and F-comp, respectively. Frequencies at which spectrum amplitudes are -10 dB smaller than the peak amplitudes are marked by circles. The gaps between the -10 dB frequencies are indicated by horizontal lines.

Data in the interval 12 ~ 145.6 ms were subjected to spectrum analysis. Classical spectrum analysis (e.g., the periodogram) was not used because of its poor frequency resolution: in our case, the resolution is about 1000 / (145.6-12) ms or ~7.48 Hz (i.e., larger than the behavioral FDLs). As an alternative, we chose the autoregressive (AR) spectral estimator, which has high resolution. The approximate resolution of AR spectral estimator is given in equation (1),

(1) $$\delta f_{\text{AR}}=\frac{1.03}{p{\left[\eta \left(p+1\right)\right]}^{0.31}}$$

where *η* is the SNR of one sinusoid, *p* is the order of the AR model [29] and *pη* > 10. The AR model parameters were estimated using the modified covariance method and *p* was set to 32. Since the minimum SNR was 4 dB, *η* ≥ 4 and the frequency resolution of the AR spectrum *δf*_*AR*_ < 0.007Hz.One example of AR-spectrum estimation is shown in Figure 1. The highest spectral peak indicates the frequency at which the FFR has most of its energy, i.e., its “characteristic frequency”, corresponding to the stimulus frequency. The -10 dB frequency points at which the spectrum amplitude is 10 dB lower than the peak amplitude were extracted. To separate FFRs to tones with different frequencies, the spectral peaks should be sufficiently narrow to prevent overlap at the -10 dB frequencies. Then, the “-10 dB frequency gap” was calculated by subtracting the higher -10 dB frequency of F_ref from the lower -10 dB frequency of F_comp, to estimate the separation between spectral peaks [see Figure 2(A)].

#### *Autocorrelation*

Autocorrelation (ACF) can be used to detect periodicity within a signal. After preprocessing, autocorrelation was performed by making a copy of the FFR signal and shifting it forward in time. For discrete signal representation, the entire signal is *x*(*n*), *n* = 1 ~ *N*. N is the total number of sampling points. The autocorrelation function is computed as equation (2).

(2) $$r\left(m\right)=\frac{\sum_{n=1}^{N}x\left(n\right)x\left(n-m\right)}{\sum_{n=1}^{N}x^{2}\left(n\right)},m=0^{\sim }N-1$$

The first peak in the normalized autocorrelation function at a non-zero lag reflects the dominant periodicity. Autocorrelation can also be used to estimate the characteristic frequency of the FFR, calculated as 1/*d*, where *d* is the time shift that yields a local maximum, representing the period of the FFR. *d* was estimated as the mean of the first ten inter-peak intervals, thus improving the accuracy of calculation.

Only FFRs evoked by F_ref were used to calculate FFR frequency tracking accuracy, since F_ref across all subjects and all experiment conditions were the same (140 Hz). The FFR signal was windowed into 30-ms bins with a 1-ms step shift of the window. The time lag of the maximum ACF peak in the *i*_*th*_ bin was ***τ***_***i***_. The RMS of differences between the time lags and the stimulus periodicity across all time bins serves as a measure of FFR frequency tracking accuracy, calculated as:

(3) $$\mathrm{\Delta a}={\left[\frac{\sum_{i=1}^{Q}{\left(\tau_{i}-\frac{1}{f}\right)}^{2}}{Q}\right]}^{\frac{1}{2}}$$

where *Q* was the total number of time bins and *f* was the frequency of stimulus. Higher Δa values indicated poorer FFR frequency tracking accuracy results [27].

### Statistical analysis

Statistical analysis was conducted with IBM SPSS Statistics 20.0 software. Levene’s Test for Equality of Variances and Shapiro-Wilk test for normality were applied for each statistical analysis except for non-parametric test.

Linear regression was used to assess the relationship between FFR measures and the stimulus frequency difference. The characteristic frequencies (FFR_F_ref and FFR_F_comp) of responses evoked respectively by the reference frequency, F_ref, and the comparison frequency, F_comp, were calculated in the frequency domain by AR spectrum estimation. The linear regression analyses were performed between the stimulus frequency difference (FD = F_comp-F_ref), the independent variable, and the FFR frequency difference (AR_FD = FFR_F_comp-FFR_F_ref), the dependent variable. In the case of autocorrelation method, the FFR period difference (ACF_PD = FFR_d_comp-FFR_d_ref) was taken as the dependent variable. A comparison of the regression results of 100% FDL condition and 75% FDL condition allowed ascertaining the correspondence between changes in the FFR measures and changes in the frequency difference between the tone stimuli.

Paired-Sample T tests were carried out to compare the differences on FFR frequency tracking accuracy in the 100% and 75% FDL conditions. For the comparison of 50% FDL condition and 75% FDL condition, non-parametric statistics Wilcoxon signed ranks tests were used.

## Results

### FFR representation of frequency difference

Figure 1A shows the waveform of the FFR evoked by a 140-Hz tone (the reference frequency), as well as data preceding stimulus onset. Figure 1(B) shows the AR spectrum (blue trace) computed from the 12 ~ 145.6 ms interval of Figure 1(A), which includes a peak at 140.3 Hz not present in the background-noise spectrum (dotted line).The FFR correlates of frequency differences were assessed by comparing the FFRs evoked by F_ref and F_comp in the spectral and time domains (Figure 2). Discrimination between individual frequencies may be inferred from the absence of overlap between the FFR spectra for the two stimuli and, in the time domain, from the difference between the FFR ACFs. Figure 2 illustrates the FFRs for one subject whose FDL was 5.63 Hz. The top and bottom rows, respectively, illustrate results for the 100% and 75%FDL conditions. The reference frequency F_ref was 140 Hz and the comparison frequencies F_comp were 145.63 Hz and 144.22 Hz. The left- and right-side panels, respectively, show the AR spectra and the FFR ACFs. As shown in Figure 2(A), the FFR spectra reach peaks at 140.57 Hz and 146.11 Hz and do not overlap at their -10 dB frequencies (where the -10 dB frequency gap between the spectra is 4.81 Hz). The spectra in panel B have peaks at 140.26 Hz and 144.24 Hz and a -10 dB frequency gap of 2.34 Hz. Graphs in the right illustrate the ACFs of the FFRs for frequencies F_ref and F_comp. The periods of the two FFR signals are 7.12 and 6.85 ms for F_ref and F_comp, respectively, in panel C and 7.12 and 6.93 ms in panel D.

### Comparison of 100% FDL and 75% FDL conditions

**Figure 3:**
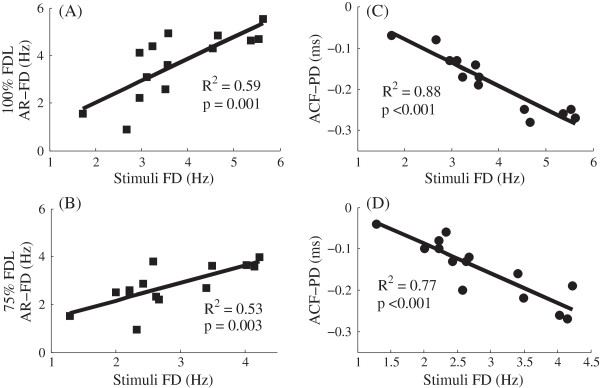
**Linear regressions between FFR measures of FD and stimulus FD.** Results for the 100% and 75% FDL conditions are shown in the top and bottom panels, respectively. FFR frequency differences measured from AR spectra (AR_FD) are shown in the left panels **(A, B)**. FFR period differences measured from ACFs (ACF_PD) are shown in the right panels **(C, D)**.

Linear regression analyses of the FFR results for the 100% and 75% FDL conditions are shown in Figure 3. The differences between the characteristic frequencies of the FFRs evoked by paired tones (AR_FD) were used for linear regression analysis. Correlations between AR_FD and the stimulus FD were significant for both the 100% and the 75%FDL conditions [(A): R^2^ = 0.59, p = 0.001; (B): R^2^ = 0.53, p = 0.003)]. Thus, it is clear that FFRs can detect the stimulus frequency differences for these two conditions. Panels C and D show that the differences between autocorrelation time lags (ACF_PD) were also significantly correlated with stimulus frequency differences [(C): R^2^ = 0.88, p < 0.001; (D): R^2^ = 0.77, p < 0.001)]. To conclude, when subjects can detect a frequency difference behaviorally in 100% FDL condition, the same difference is also reflected by the FFR; and the FFR could also reflect a difference that was not behaviorally detectable in 75% of FDL condition. The results of FDL and FFR frequency tracking for the 100% and 75% FDL conditions in all subjects are listed in Table 1. Two tailed paired-sampled T tests were performed comparing tracking accuracy Δa in the two conditions. For 13 subjects with available data from both conditions, no significant differences were found [t (12) = 1.87, p = 0.086]. Besides, subjects S1 ~ S12 in 100% FDL were paired with subjects S4 ~ S15 in 75% FDL to achieve similar stimulus FD for the two conditions, and still no significant difference existed [t (10) = 1.32, p = 0.215]. In other words, FFRs detected frequency differences smaller than the FDLs because of FFRs’ accurate phase-locking to individual frequencies.

**Table 1:** Behavioral FDL and FFR frequency tracking for all subjects

***Subject***	***FDL***	***100% FDL condition***			***75% FDL condition***		
		***AR_FD***	***ACF_PD***	**∆** ***α (ms)***	***AR_FD***	***ACF_PD***	**∆** ***α (ms)***
***No.***	***(Hz)***	***(Hz)***	***(ms)***		***(Hz)***	***(ms)***	
S1	1.72	1.55	-0.07	0.219	1.51	-0.04	0.158
S2	2.67	0.88	-0.08	0.112	2.51	-0.10	0.081
S3	2.96	4.12	-0.13	0.206	2.48	-0.08	0.138
S4	2.96	2.21	-0.13	0.147	2.60	-0.10	0.086
S5	3.11	3.09	-0.13	0.131	0.96	-0.06	0.131
S6	3.24	4.39	-0.17	0.126	2.87	-0.13	0.127
S7	3.44	None	None	None	3.81	-0.20	0.226
S8	3.51	2.57	-0.14	0.365	2.34	-0.13	0.102
S9	3.57	3.6	-0.19	0.107	2.20	-0.12	0.153
S10	3.58	4.93	-0.17	0.195	None	None	None
S11	4.54	4.31	-0.25	0.127	2.68	-0.16	0.083
S12	4.66	4.84	-0.28	0.128	3.62	-0.22	0.103
S13	5.37	4.63	-0.26	0.083	3.64	-0.26	0.148
S14	5.54	4.69	-0.25	0.297	3.58	-0.27	0.172
S15	5.63	5.54	-0.27	0.088	3.97	-0.19	0.094
Mean	3.77	3.67	-0.18	0.17	2.77	-0.15	0.13
Standard deviation	1.10	1.35	0.07	0.08	0.85	0.07	0.04

AR_FD represents the difference between characteristic frequencies of FFRs evoked by F_ref and F_comp using AR algorithm, while ACF_PD represents the period difference using ACF algorithm. ∆*α* represents FFR frequency tracking accuracy calculated as equation (3).

### Comparison of 75% FDL and 50% FDL condition

**Figure 4:**
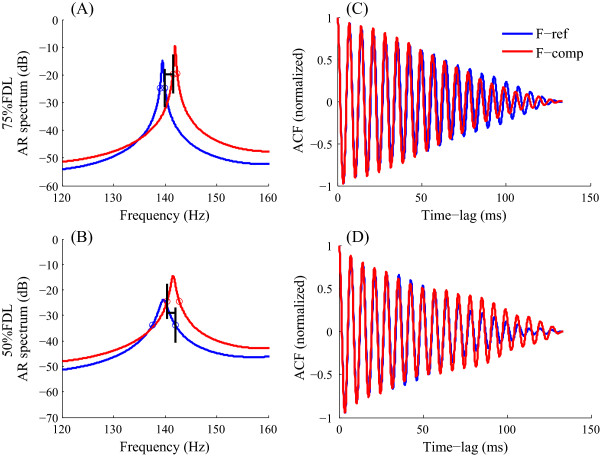
**FFR spectra and ACFs for 75% FDL and 50% FDL conditions.** Data are from the first pair in Table 2 (Subject S2 in 75% FDL condition and Subject S8 in 50% FDL condition). Left-side panels **(A, B)** show the spectra and right-side panels **(C, D)** show the corresponding ACFs. Top to bottom panels, respectively, illustrate results in the 75% and 50% FDL conditions. Other conventions are the same as for Figure 2.

Six subjects whose 50% FDL was similar to the 75% FDL of other subjects were selected to participate in the 50% FDL condition, as listed in Table 2. Subjects with FDLs in the 50% FDL condition were paired with other subjects whose FDL in the 75% FDL condition were similar. For example, subject S12 in the 50% FDL condition was paired to subject S5 in the 75% FDL condition. Comparisons of FFRs differences are shown in Figure 4 for Subject S2 (75% FDL condition) and Subject S8 (50% FDL condition). In the 75% FDL condition, two FFRs evoked by 140 Hz and 142 Hz showed spectral peaks at 139.48 and 141.93 Hz, respectively. The gap between -10 dB frequencies of the two spectra is 2.37 Hz. For the 50% FDL condition, the spectra in panel B with peaks at 139.69 and 141.56 Hz largely overlapped and the -10 dB frequency gap was -1.59 Hz. Panels in the right illustrate the ACFs for the FFRs evoked by F_ref and F_comp. The FFR periods in the 75% FDL condition (panel C) are different, 7.15 and 7.05 ms, whereas the FFR periods for the 50% FDL condition (panel D) are nearly the same (7.12 and 7.1 ms). Wilcoxon signed ranks test showed the FFR frequency tracking accuracy (see Δa in Table 2) was significantly poorer in the 50% FDL condition than the 75% FDL condition (z = -2.201, p = 0.028). Thus, FFRs detected the frequency difference in the 75% FDL condition, but not in the 50% FDL condition, even though the stimulus frequency differences were similar in both conditions.

**Table 2:** Paired subjects in two FFR conditions with same stimulus FD

***Pair NO.***	***75% FDL condition***			***50% FDL condition***		
	***Subject***	***Stimulus***	**∆** ***α***	***Subject***	***Stimulus***	**∆** ***α***
	***NO.***	***FD (Hz)***	***(ms)***	***NO.***	***FD (Hz)***	***(ms)***
1	S2	2	0.081	S8	1.76	0.15
2	S3	2.22	0.138	S9	1.79	0.163
3	S4	2.22	0.086	S11	2.27	0.319
4	S5	2.33	0.131	S12	2.33	0.176
5	S8	2.63	0.102	S15	2.82	0.156
6	S9	2.68	0.153	S13	2.68	0.166
Mean		2.35	0.12		2.28	0.19
Standard deviation		0.24	0.03		0.40	0.06

## Discussion

Frequency-difference discrimination in humans is usually tested using psychophysical methods, which depend on the entire auditory system. Behavioral just noticeable frequency differences above 500 Hz increase in proportion to the reference frequency at a rate of 0.2%; the just noticeable frequency difference at lower frequencies is about 1 Hz [2]. In the present study, behavioral FDLs varied from 1.72 to 5.63 Hz with mean (standard deviation) being 3.79 (1.18) Hz, or 1.23% ~ 4.02% of the reference frequency 140 Hz with mean relative FDL being 2.71%.

Physiological representations of frequency difference were measured using FFRs evoked by pairs of tones. In the 100% FDL condition, when subjects detected a frequency difference behaviorally, the FFRs also reflected that frequency difference. In the 75% FDL condition, when the subjects could not recognize the frequency difference, the FFR still detected a difference. This indicates that the FFR correlate of frequency discrimination is smaller than the FDL, probably due to additional factors such as attention and short-term working memory. Musicians might be better in short-term working memory so that yielded significant better performance both neutrally and behaviorally than non-musician English speakers [15]. Besides, Marmel et al. found that behavioral FDL was not only affected by FFR phase-locking but also absolute thresholds [21]. Our results are consistent with a study by Clinard et al. which also demonstrated that FFRs could detect smaller frequency differences than the FDLs [8]. In Clinard et al. study, the ratio of FFR phase coherences between the two frequencies (for example, 998:1000 Hz) was related to behavioral FDL (at 1000 Hz), but the ratio was not significantly predictive of behavioral FDL [8].

In a previous study on frequency discrimination of auditory cortex, MMNs were elicited by frequency changes of 2.5%, 5%, 10% and 20% across a frequency range of 250 ~ 4000 Hz [30]. Larger frequency differences elicited MMNs with larger amplitudes and shorter latencies. In other words, it was more accurate for the cortex to distinguish larger frequency difference. Consistently, in our study FFR phase-locking was more accurate when evoked by stimuli with larger frequency difference. The accuracy of FFR frequency tracking was similar for frequency differences equal to 100% FDL and 75% FDL, but FFR frequency tracking accuracy significantly decreased in the 50% FDL condition. We presume that the strength of the FFR representation of frequency differences covaried with the FDLs, which is consistent with results of some previous studies [15,17-21].

## Conclusion

We studied individual frequency-difference discrimination by recording brainstem FFRs and measuring behavioral FDLs in the same subjects. In the present study, discrimination of FFRs to individual frequencies was used to reflect the stimulus frequency difference. Our results showed that FFR can represent a smaller frequency difference than the behavioral FDL.

## Abbreviations

ABR: Auditory brainstem response; AR: Autoregressive; ACF: Autocorrelation function; FD: Frequency difference; FDL: Frequency difference limen; FFR: Frequency following response; F_ref: The reference frequency; F_comp: The comparison frequency; PD: Period difference; SPL: Sound pressure level; SNR: Signal to noise ratio; RMS: Root mean square.

## Competing interest

The authors report no declarations of interest.

## Authors’ contributions

QG designed the experiments and was responsible for revising this manuscript. This study originated from QG’s idea and problems were solved under her directions. QX initiated and conceived the algorithm, analyzed the data and drafted this paper’s manuscript. All authors read and approved the final manuscript.
